# Nanophotonic Enhanced Chiral Sensing and Its Biomedical Applications

**DOI:** 10.3390/bios14010039

**Published:** 2024-01-12

**Authors:** Fei Wang, Xue Wang, Xinchao Lu, Chengjun Huang

**Affiliations:** 1 Institute of Microelectronics of the Chinese Academy of Sciences, Beijing 100029, China; wangfei2020@ime.ac.cn; 2 University of Chinese Academy of Sciences, Beijing 100049, China; 3 Institute of High Energy Physics, Chinese Academy of Sciences, Beijing 100049, China; wangxue@ihep.ac.cn

**Keywords:** chiral sensing, nanophotonics, circular dichroism, surface plasmons

## Abstract

Chiral sensing is crucial in the fields of biology and the pharmaceutical industry. Many naturally occurring biomolecules, i.e., amino acids, sugars, and nucleotides, are inherently chiral. Their enantiomers are strongly associated with the pharmacological effects of chiral drugs. Owing to the extremely weak chiral light–matter interactions, chiral sensing at an optical frequency is challenging, especially when trace amounts of molecules are involved. The nanophotonic platform allows for a stronger interaction between the chiral molecules and light to enhance chiral sensing. Here, we review the recent progress in nanophotonic-enhanced chiral sensing, with a focus on the superchiral near-field and enhanced circular dichroism (CD) spectroscopy generated in both the dielectric and in plasmonic structures. In addition, the recent applications of chiral sensing in biomedical fields are discussed, including the detection and treatment of difficult diseases, i.e., Alzheimer’s disease, diabetes, and cancer.

## 1. Introduction

Chirality, referred to as handedness manifesting the asymmetrical nature of matter, is the property that ensures that an object cannot overlap with its mirror counterpart through rotation and translation. Chirality is ubiquitous in nature [1], ranging from microscopic molecules such as amino acids and DNA, to animals and plants, and even to macroscopic celestial motion. The chirality of molecules is of vital significance in biomedical applications, which is demonstrated by the shocking Thalidomide incident. Molecules possessing the same composition with a different chirality are called enantiomers, i.e., R-type and S-type thalidomide. R-type thalidomide is a sedative for relieving morning sickness during pregnancy, while S-type thalidomide, the other enantiomer, has obvious teratogenic effects. Without chirality sensing, the S-enantiomer was misused as a sedative for pregnant women, which led to the birth of many malformed fetuses [2]. After the Thalidomide incident, enantiomer identification in drug synthesis became necessary for the pharmaceutical industry.

Historically, the initial observation of chiral–optical activity can be traced back to 1811, when Arago noted the colors in the sunlight that passed through quartz between two polarizers [3]. Then, chirality-sensing methods were designed to identify the chirality of molecules, which commonly rely on dichroism or birefringence. As the chiral molecules usually exhibit polarization-dependent optical properties, optical analytical techniques are indeed well-suited to sensing and characterizing chirality [2,3,4]. Interacting with circularly polarized light fields, the molecular chirality is either manifested as the differential absorption of left- and right-handed circularly polarized light (CPL), referred to as circular dichroism (CD), or indicated by the rotation of the polarization plane of the linearly polarized light, termed optical rotatory dispersion (ORD). CD spectroscopy is a routine characterization tool to investigate the chiral properties of molecules, particularly proteins and nucleic acids. As an illustration of this, the secondary and tertiary structures of proteins display distinctive CD responses in the ultraviolet (UV) spectra, rendering CD spectroscopy a robust technique for the identification, characterization, and evaluation of protein conformations. However, owing to the mismatch in scale between the helical pitch of atoms in molecules and the wavelength of light, the CD signal of chiral molecules is naturally faint, and the sensitivity of chiral-optical spectroscopies is inherently limited. In order to obtain adequate signal-to-noise ratios, substantial amounts of chiral molecules are necessary, which is a practical limitation for scarce or expensive samples.

**Figure 1:**
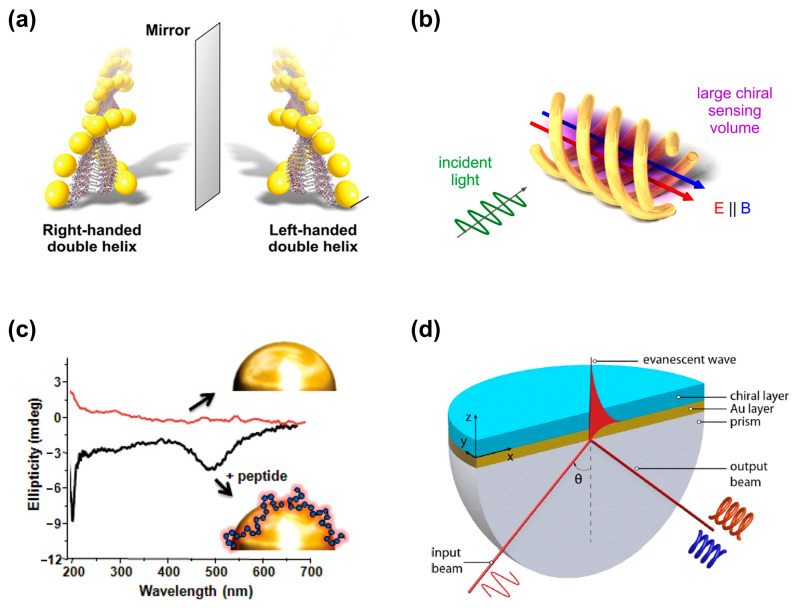
Nanophotonic platforms for enhanced chiral detection and recognition. (**a**) Peptide-based self-assembly of enantiomeric gold nanoparticle double helices. Adapted with permission from ref [8]. Copyright 2013 American Chemical Society. (**b**) Three-dimensional chiral plasmonic metal structures generating super chiral near-fields. Reprinted with permission from ref. [9]. Copyright 2014 American Chemical Society. (**c**) Plasmon-coupled circular dichroism (PCCD). Adapted with permission from ref. [10]. Copyright 2011 American Chemical Society. (**d**) Surface plasmon resonance based on Kretschmann configuration for chiral recognition. Adapted with permission from ref. [11]. Copyright 2019 American Chemical Society.

Recent progress in nanophotonics offers solutions for enhancing chiral–optical interactions. Benefiting from the potent interactions between light and matter within nanophotonic resonators, chiral molecules are detected at extremely low concentrations [4]. For instance, metallic nanoparticles manifest robust localized surface plasmon resonance (LSPR) in the visible or near-infrared (IR) spectral range, rendering them outstanding candidates for enhancing the interactions between light and chiral matter [5,6,7]. In this review, we discuss four typical nanophotonic platforms for enhancing chirality-sensing and their potential biological applications. As shown in Figure 1, the common detection platforms mainly include: (a) the bottom-up self-assembly of achiral metal nanoparticles into chiral structures; (b) nanostructures to produce a super chiral near-field, thereby enhancing chiral–optical interactions; (c) the coupling between chiral molecules and noble metal nanoparticles to generate enhanced plasmon-coupled CD (PCCD); (d) propagating surface plasmon resonance to detect chiral molecules. Meanwhile, the applications of nano-optical chiral platforms in some difficult disease diagnoses and treatments, such as Alzheimer’s disease, diabetes, and cancer, were also reviewed.

## 2. Nanophotonic Platforms for Enhancing Chirality Sensing

### 2.1. Structural Chirality Based on Self-Assembly

**Figure 2:**
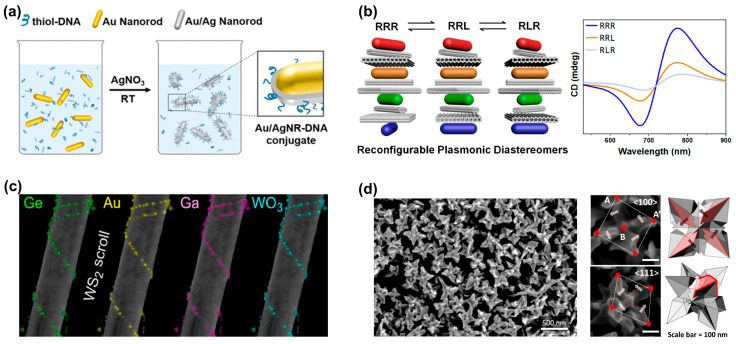
Generating structures with chiral characteristics through self-assembly processes and direct chiral synthesis. (**a**) Au nanorods and Au/Ag nanoroads were assembled into chiral structures through the DNA origami coupling process. Reprinted with permission from ref. [16]. Copyright 2020 American Chemical Society. (**b**) Self-assembled reconfigurable plasmonic diastereomers through DNA origami nanotechnology. Reprinted with permission from ref. [13]. Copyright 2019 American Chemical Society. (**c**) Achiral inorganic nanotubes are used as templates for the chiral assembly of nanoparticles to achieve the chiral self-assembly of various nanoparticles. Reprinted in part under the terms of a Creative Commons CC-BY 4.0 license from ref. [17]. Copyright 2023 American Chemical Society. (**d**) SEM image of chiral gold nanoparticles synthesized directly with adenine oligomers. Points A and B represent the corner points and center points of the nanoparticles, respectively. The arrows indicate the direction of chiral rotation of the nanoparticle. Reprinted in part under the terms of a Creative Commons CC-BY license from ref. [18]. Copyright 2022 Springer Nature.

During the self-assembly process, the optical properties of nanoparticles are fine-tuned via the particle size, shape, and composition, which makes self-assembly one of the most promising tools for constructing chiral macroscopic materials. Owing to their inherent structural chirality, DNA [12,13,14], peptides [8,15], and proteins are usually selected as templates to assemble achiral metal nanoparticles into chiral structures. As shown in Figure 2a, Nguyen et al. used DNA origami technology to synthesize highly stable gold-silver core-shell nanoparticles [16]. The silver shell induced more plasmonic enhancement than gold nanorods, and the bimetallic chiral assembly showed a strongly increased CD response, which holds great promise for plasmonic sensing. Wang et al. proposed self-assembled reconfigurable plasmonic diastereomers based on DNA origami nanotechnology [13]. Using a stepwise assembly strategy, they constructed plasmonic diastereomers with up to three distinguishable chiral centers. As shown in Figure 2b, each chiral center is selectively controlled to produce the desired diastereomer, resulting in a characteristic chiral optical signal. Meanwhile, they found that the chiral center of most diastereomers dominates the overall CD signal and the CD output.

Compared to DNA, amino acids, micelles, etc., the use of naturally “hard” inorganic templates provides more freedom for further processing steps or the functionalization of optically active nanoparticles [17]. In 2017, Cheng et al. suggested the utilization of silica nanohelices as a chiral template substrate for the self-assembly of gold nanoparticles through electrostatic adsorption, leading to a chiral three-dimensional superstructure composed of gold helices [19]. Similarly, employing the nanosilica helical structure as a template, Negrín-Montecelo et al. sequentially self-assembled gold and TiO nanoparticles onto the substrate, and finally achieved a composite chiral structure with photocatalytic properties [20]. In addition to silica templates, layered van der Waals materials (WS_2_) have also been used as templates for the chiral assembly of nanoparticles. Kachtík et al. found that both metallic and dielectric nanoparticles were adsorbed to the nanotube surface through the chemically active site: an unsaturated sulfur bond in WS_2_ [17]. As shown in Figure 2c, they demonstrated the assembly of various chiral nanoparticle structures based on WS_2_ nanotubes, including metals (Au, Ga), semiconductors (Ge, GaAs), and oxide nanoparticles (WO_3_).

An alternative approach for fabricating chiral plasmonic nanostructures involves directional growth during the nanoparticle synthesis process [18,21]. As shown in Figure 2d, Cho et al. introduced adenine oligomers during the synthesis of nanoparticles to induce chiral morphology evolution [18]. The synthesized chiral gold nanoparticles exhibit an asymmetric factor of up to 0.04 in the visible-light wavelength range. In addition, the disparate binding energies of various amino acid enantiomers to the gold crystal surface regulate the growth pathways of nanoparticles. Consequently, the utilization of amino acids and small peptides allows for the controlled growth of gold nanoparticles. Lee and co-workers [22] developed a method to control the chiral morphology of gold nanoparticles through the molecular interactions of amino acids or peptides with high-refractive-index surfaces. Gold nanoparticles fabricated by this method display strong chiral plasmonic optical activity (asymmetry factor of 0.2), even when randomly dispersed in solution. Halides, especially iodide and bromide ions, show preferential adsorption on specific crystal planes, thus initiating the formation of chiral structures with noble metal nanoparticles of different shapes [23]. Recently, in recognition of the influence of anisotropic seeds and the effect of halides on directional growth, Zheng et al. [24] introduced a growth strategy termed Halide-Assisted Differential Growth (HADG), which is applied to anisotropic metal nanoparticle seeds and successfully yields plasmonic metal nanocrystals characterized by distinct chiral shapes.

### 2.2. Superchiral Near-Fields

In 1964, Daniel M. Lipkin [25] first introduced a new time-even pseudoscalar, whose physical meaning was later supplemented and improved by Tang et al. [26] in 2010, i.e., optical chirality *C*, which is defined as:

(1) C≡ε02E⋅∇×E+12μ0B⋅∇×B=−ω2c2Im(E*⋅H)=−ω2c2EHcos(βiE,H)

where $\varepsilon_{0}$ and $\mu_{0}$ are the permittivity and permeability in free space, respectively, and **E** and **B** represent the time-dependent electric and magnetic fields. The angle *β_i_***_E_**_,**H**_ indicates the phased angle between *i***E** and **H**. In Equation (1), to generate a larger ***C***, the following three conditions must be satisfied: firstly, electric field **E** and magnetic field **H** must be enhanced; secondly, both **E** and **H** must have parallel components; thirdly, **E** and **H** must have a non-zero phase difference, preferably a phase difference of π/2. For CPL in free space, the optical chirality *C*_CPL_ is given by:

(2) $$C_{\mathrm{CPL}}=\pm \frac{\varepsilon_{0}\omega }{2c}{E_{0}}^{2}$$

where *E*_0_ is the electric field magnitude in the free space. The sign of *C*_CPL_ is contingent upon the handedness of CPL (LCP: +; RCP: − ). With $C/C_{\mathrm{CPL}}>1$ , a “superchiral” field is generated. The relationship between the optical chirality *C* and the rate of absorption by a chiral molecule is typically described by [26]:

(3) $$A^{\pm }=\frac{\omega }{2}(\alpha \prime \prime {\left|E\right|}^{2}+{\mu_{0}}^{2}\chi \prime \prime {\left|H\right|}^{2})\mp \frac{2}{\varepsilon_{0}}G\prime \prime C$$

In Equation (3), $A^{\pm }$ is the absorption rate of chiral molecules for LCP and RCP, respectively, and α″ is the electric polarizability. χ″ and G″ represent the imaginary components of the magnetic and chiral polarizability of the chiral molecule, respectively. According to the definition of CD, it can be expressed as:

(4) $$CD\propto A^{+}-A^{-}=-\frac{4}{\varepsilon_{0}}G\prime \prime C$$

From Equation (4), increasing the optical chirality, *C*, is an effective solution to enhance the CD magnitude of the chiral molecule detection.

#### 2.2.1. Plasmonic Nanostructures

**Figure 3:**
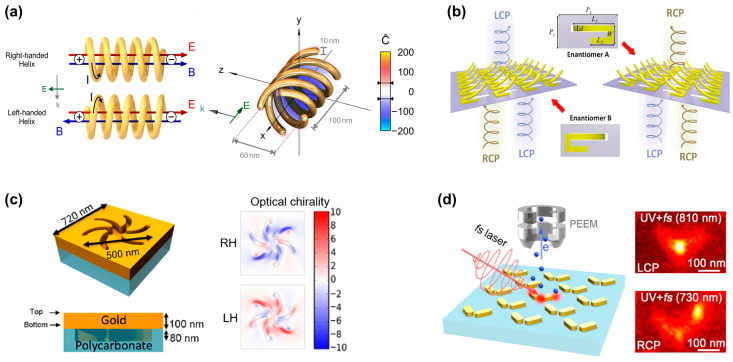
Generating superchiral fields based on plasmonic nanostructures. (**a**) Two spiral plasmonic nanoantennas with different helical orientations exhibit opposite basic modes. The structures with four helices exhibit strong superchiral near fields. Adapted with permission from ref. [9]. Copyright 2014 American Chemical Society. (**b**) Schematic of the 3D-bended metasurface. These asymmetric metasurfaces exhibit different responses to circularly polarized light in the mid-infrared range. Reprinted with permission from ref. [27]. Copyright 2021 John Wiley and Sons. (**c**) The schematic diagram of 2D planar chiral shuriken nanostructure and its optical chirality (RH: right-handed, LH: left-handed). Adapted in part under the terms of a Creative Commons CC-BY 4.0 license from ref. [28]. Copyright 2022 American Chemical Society. (**d**) Gold nanorod dimers with chiral optical responses. Reprinted in part under the terms of a Creative Commons CC-BY-NC-ND 4.0 license from ref. [29]. Copyright 2021 American Chemical Society.

Owing to their intrinsic chirality, chiral plasmonic substrates are prone to generating a strong optical chirality *C*, which makes them competitive candidates for chiral sensing based on superchiral field. However, the left- and right-handedness exist simultaneously in many structures and cancel each other out, ultimately showing a weak overall near-field optical chirality. To overcome this challenge, Schäferling et al. [9] proposed a 3D chiral substrate composed of multiple helices, as shown in Figure 3a, which greatly enhances optical chirality *C* over a larger sensing volume. Specifically, the fundamental mode of the helical plasmonic nanoantenna exhibits non-orthogonal electric and magnetic dipole moments. Inside the structure, the electric field vector (red field) and magnetic field vector (blue field) are mainly parallel, resulting in a non-zero optical chirality. Changing the chirality of the structure flips the relative direction of the field vectors, and thus shifts the chirality near field. To achieve both a strong chiral near field and good coupling to external fields, additional helices are added. As shown in the diagram on the right side of Figure 3a, the optical chirality inside the entire structure is right-handed (the value of *C* is negative). As the nanostructure is difficult to manufacture, the authors enlarged the size of the helix to 12.6 μm, which is experimentally realized by 3D direct laser writing.

Traditional methods for constructing 3D structures often involve 3D printing or the assembly of multiple layers [30], which lead to challenges associated with large feature sizes, extended processing times, and intricate procedures. Alternatively, various origami methods that address the limitations associated with folding and bending 2D films have been developed, providing innovative solutions for complex three-dimensional structures from initially flat materials. As shown in Figure 3b, Gu’s group [27] reported an asymmetric bent split ring resonator array constructed from tensile stress, which is fabricated by focused ion beam–matter interaction. As the folding angle of the split ring increases, the CD signal gradually increases and reaches the maximum at 60°. At the same time, the calculation results of the near-field distribution and electric/magnetic dipoles indicate that the giant CD originates from the cross-coupling of the parallel components of the electric and magnetic dipole moments.

Two-dimensional planar chiral nanostructures are also known as pseudochiral nanostructures but exhibit hand-dependent responses to circularly polarized light at normal incidence. These structures acquire chirality through chiral-substrate-induced symmetry breaking or manufacturing defects, which eliminates the need for 3D characteristics, thus simplifying the fabrication process [31]. Figure 3c shows a typical planar chiral nanostructure [28]. The simulation results indicate the significantly enhanced near-field optical chirality.

In addition to the optical chiral characteristics observed in the chiral structures mentioned above, optical chirality is also generated in achiral structures. In achiral structures, chirality results from symmetry breaking, including oblique illumination, structural plane tilt, etc. As shown in Figure 3d, Misawa’s group investigated two orthogonally oriented gold nanorods to produce giant handedness with obliquely incident LCP and RCP light [29]. The results show that pattern excitation in nanostructures plays a crucial role in the generation of chiral optical responses. Two basic dipole modes, i.e., antisymmetric and symmetric modes, are induced in the gold nanorod dimer by the oblique incidence of LCP and RCP light and generate chirality. Surprisingly, optical chirality is observed in achiral nanostructures even under symmetric illumination. Horrer et al. [32] revealed the optical chirality inside highly symmetric plasmonic metamolecules. Taking a trimer composed of three gold nanodisks arranged in an equilateral triangle, they found that the local optical chirality originates from the near-field interference coupling between plasmon modes generated by a single nanodisk.

#### 2.2.2. Dielectric Nanostructures

**Figure 4:**
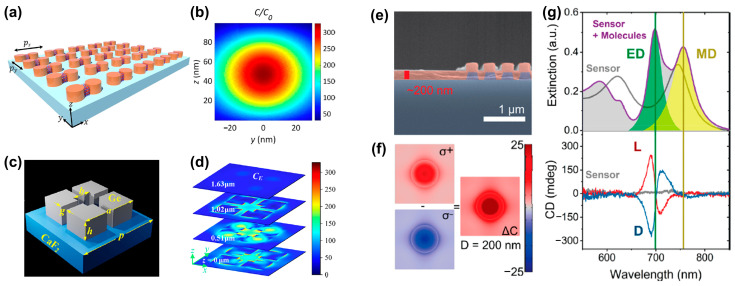
Superchiral field generation in dielectric structures. (**a**) A dimer array of two silicon nanocylinders (diameter D = 140 nm, height h = 100 nm). (**b**) Distribution of normalized optical chirality in the y-z plane of the structure (middle of the gap region) at the resonance wavelength. Adapted with permission from ref. [34]. Copyright 2022 American Chemical Society. (**c**) Schematic diagram of the unit cell of a metasurface structure composed of an achiral Ge tetramer nanoresonator array, where *a* = 3.0682 μm, *b* = 1.0794 μm, *h* = 1.0275 μm, *g* = 0.3901 μm and *p* = 4.581 μm. (**d**) Optical chirality distribution on sections at different heights. Adapted with permission from ref. [35]. Copyright 2023 American Chemical Society. (**e**) Scanning electron micrograph of a cross-section of a Si sensor (purple) coated with a ∼200 nm thick chiral phenylalanine coating (red) [39]. (**f**) Near-field optical chiral enhancement distribution around a dielectric cylinder [39]. (**g**) Experimental extinction spectra (top) and CD spectra (bottom) of 120 × 120 μm bare and coated sensors. Adapted with permission from ref. [39]. Copyright 2020 American Chemical Society.

In addition to plasmonic nanostructures, dielectric nanostructures are also used for generating superchiral fields. High-index dielectric materials, i.e., silicon, intrinsically support electrical and magnetic resonances simultaneously, which makes them attractive for chiral sensing. Compared with plasmonic metallic structures, although dielectric nanomaterials have lower optical losses [33], their local electric field enhancement is weaker. Rui’s group [34] proposed a metasurface consisting of a dimer array of silicon nanocylinders in a square lattice, as shown in Figure 4a. Unlike traditional structures, which require circularly polarized light to excite the superchiral near field, the researchers found that illumination with off-axis polarization leads to superchiral localized hot spots in the gaps of the dimer structure. Meanwhile, the near-field electric and magnetic fields of a single resonator are significantly modified by coupling with adjacent resonators, resulting in stronger local field enhancement. The optical chiral field is enhanced by more than 300 times, as shown in Figure 4b. Recently, Altug’s [35] team proposed a metasurface based on an achiral Ge tetramer resonator array (Figure 4c). Due to the Mie resonance, the Ge resonator achieves strong magnetic field and electric field enhancement, thereby achieving superchiral near-fields. Figure 4d shows the optical chirality distribution at different cross-section positions. The optical chirality field is usually positive on all cutting planes, which is very important for enhanced chiral sensing. As far as the fabrication is concerned, since the critical dimensions of the structure exceed 300 nm, it is expected to be compatible with wafer-level nanomanufacturing processes [36], such as deep ultraviolet lithography [37], and nanoimprinting [38].

To explore how dielectric nanoresonators effectively distinguish molecular enantiomers and analyze the impact of detuning between electric dipole (ED) and magnetic dipole (MD) resonances on the CD signal, J. García-Guirado et al. [39] discussed amorphous silicon nanocylinder dielectric nanoresonators, as shown in Figure 4e. The simulation results in Figure 4f show that the optical chirality is enhanced by more than 25 times. As shown in Figure 4g, the maximal value of the CD spectrum appears close to the electric dipole resonance peak, while the minimal value is situated at the position of the magnetic dipole resonance peak. Therefore, they claimed that the CD spectral response is mainly dominated by electric dipoles.

#### 2.2.3. Plasmonic and Dielectric Hybrid Platform

**Figure 5:**
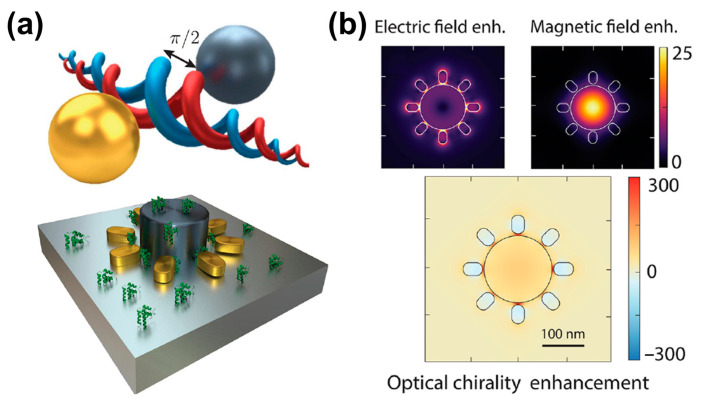
Plasmonic and dielectric hybrid platform for the generation of superchiral field. (**a**) Hybrid metal–dielectric structure of a silicon disk surrounded by gold nanorods. (**b**) Electric and magnetic field distribution under the resonance wavelength. The figure below shows the optical chirality enhancements. Adapted in part under the terms of a Creative Commons CC-BY-NC-ND 4.0 license from ref. [40]. Copyright 2021 American Chemical Society.

Since metallic and dielectric nanoparticles provide strong electric and magnetic resonance, metal–dielectric hybrid structures are expected to achieve strong near-field optical chirality. To obtain optimal optical chirality, in addition to maximizing the resonance intensity, the resonances must also spectrally coincide. Simultaneously, the components of the electric and magnetic fields must be parallel and possess a π/2 phase shift, as well as overlap in space, as shown in Figure 5a. To satisfy the conditions for optimal optical chirality, Mohammadi et al. [40] proposed a hybrid composition of metallic (gold) and high-refractive-index dielectric (silicon) particles, in which both metallic and dielectric nanoparticles provide electric and magnetic resonances. Since the helicity of the incident field is retained, the phase coefficient is perfect, and the scattered light with a phase difference of π/2 between the electric and magnetic field components is ensured, a more perfect superchiral field is produced. To make up for the small size limitation of simple dimer chiral hotspots, they proposed a hybrid metal-dielectric metasurface to perform practical chiral sensing by utilizing multiple chiral hotspots, as shown in Figure 5a bottom. The simulation results in Figure 5b show that the coupled electrical resonances in the nanorod and disk provide a strong electrical hotspot at the resonance wavelength of the disk. These spectrally overlapping resonances provide electric and magnetic fields with π/2 phase shift, resulting in a 300-fold enhancement of optical chirality in each nanogap.

### 2.3. Plasmon-Coupled Circular Dichroism

**Figure 6:**
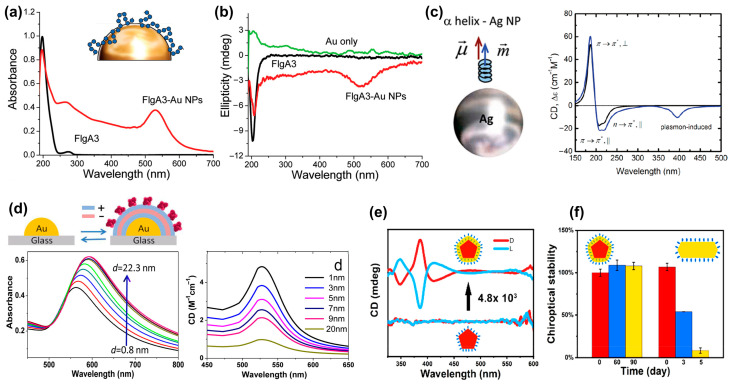
Plasmon coupled circular dichroism. (**a**) UV-visible spectra of FlgA3 peptide and FlgA3-gold nanoparticles. Inset: Illustration of the surface of a gold nanoparticle linked to the FlgA3 peptide via noncovalent interactions [10]. (**b**) Comparison of the CD spectra of three different structures: Au particles, FlgA3 peptide and FlgA3-Au particles in the ultraviolet and visible light regions, respectively. Adapted with permission from ref. [10]. Copyright 2011 American Chemical Society. (**c**) Model of interaction between Ag NPs and α-helices. Calculated CD spectral signals of α-helix (black line) and NP molecular complex (blue line). Reprinted with permission from ref. [41]. Copyright 2010 American Chemical Society. (**d**) Absorbance and CD spectra as a function of distance between gold islands and chiral molecules. Reprinted with permission from ref. [42]. Copyright 2013 American Chemical Society. (**e**) CD spectra of L- or D-Cys-embedded Ag NPs and L- or D-Cys functionalized Ag NPs [43]. (**f**) chiroptical stability of L- or D-Cys-embedded Ag NPs and L- or D-Cys functionalized Ag NPs. Reprinted with permission from ref. [43]. Copyright 2023 American Chemical Society.

PCCD is another important method used to identify chiral molecules, where the CD originates from the Coulomb interaction (dipole and multipole) between the chiral molecule and the achiral plasmonic structures [41]. Figure 6a shows a comparison of the absorption spectra of gold nanoparticles functionalized with and without FlgA3 peptides [10]. Compared with the absorption peak of the FlgA3 peptide located in the ultraviolet band, the FlgA3 peptide–Au particle complex produced a new absorption peak at ~520 nm, resulting in the repeatable characteristic peaks in the visible light band of CD spectrum. Figure 6b shows the comparison results of CD spectra of three different structures: FlgA3 peptides, an Au particle, and a FlgA3 peptide–Au particle complex. This newly induced CD peak is mainly attributed to the electronic interaction between the chiral peptide and metal electrons [10].

In 2010, Govorov et al. theoretically studied the optical chiral response of nanoscale composites composed of chiral molecules and achiral nanocrystals [41]. As shown in Figure 6c, a theoretical model of the interaction between Ag NPs and α-helices was constructed, which reveals the existence of two primary mechanisms governing PCCD effects. The first one involves a plasmon-induced electromagnetic field within the chiral molecule. The second one is attributed to the light absorption exhibited by the plasmonic nanostructure–molecule complex, arising from the chiral current within the nanostructure induced by the dipoles of the chiral molecules. Subsequently, by regulating the distance between plasmonic gold island and chiral molecules, Maoz et al. [42] investigated the relationship between molecule–metal distance and the strength of PCCD, as shown in Figure 6d.

Recently, the stability of PCCD-based plasmonic nanoparticles has also attracted widespread attention. In most PCCD systems, chiral molecules are gathered on the surface of plasmonic nanoparticles through chemical adsorption, and the desorption of molecules undoubtedly seriously affects the stability of the entire coupled system. Wei et al. found that this problem can be significantly improved by embedding chiral molecules into Ag NPs [43]. As shown in Figure 6e,f, the coupling structure not only exhibits a CD signal enhancement of up to 4800 times but also has excellent stability, with almost no change in the CD signal after 3 months.

### 2.4. Surface Plasmon Resonance Platform

**Figure 7:**
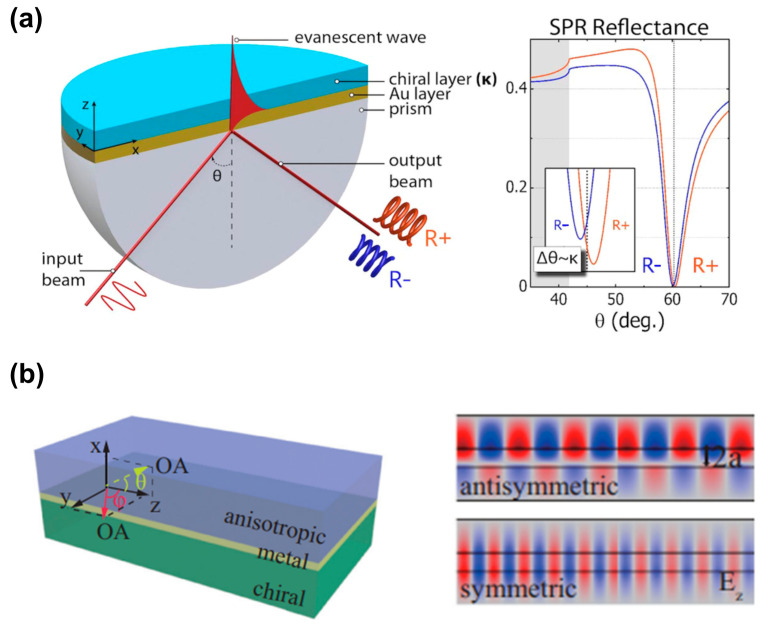
Chiral sensing scheme based on propagation surface plasmon resonance. (**a**) Schematic of the surface plasmon resonance experimental device based on the Kretschmann configuration, and the reflection spectrum curve when different chiral molecular layers exist. Reprinted with permission from ref. [11]. Copyright 2019 American Chemical Society. (**b**) Schematic diagram of an anisotropic metal chiral waveguide with the optical axis (OA) on the yz or xz plane. The right figure indicates the antisymmetric modes and symmetric modes supported by the chiral waveguide. Reprinted with permission from ref. [44]. Copyright 2020 American Physical Society.

Recently, researchers have discovered that macroscopic platforms based on surface plasmons are also expected to recognize and detect chiral molecules. Droulias and Bougas [11] proposed an angle-resolved chiral surface plasmon resonance (SPR) scheme based on the Kretschmann configuration, as shown in Figure 7a, which detects the absolute chirality (handedness and magnitude) of chiral samples. As SPR reflectance is sensitive to both the real and imaginary parts of the refractive index of the chiral samples, the reflection spectrum curve in Figure 7a indicates that the excitation angle of SPR is different for various chiral layers. Therefore, the excitation angle is used to characterize the chiral molecules. Although this work only contains theoretical calculations and simulation results, it provides a simple idea for the identification of chiral enantiomers. Similarly, as shown in Figure 7b, Zhang et al. [44] proposed a simple waveguide structure built from anisotropic birefringent crystal–metal–chiral media, revealing the chirality-dependent dispersion and propagation properties of surface plasmon polaritons. The theoretical results demonstrate that this structure discriminates between the magnitude and sign of the real and imaginary parts of chirality parameters.

Recently, the concepts of transverse spin angular momentum and Bellinfant spin momentum of evanescent waves have attracted widespread attention [45,46]. As a type of evanescent wave, surface plasmons also carry an inherent transverse spin angular momentum, which is locked in its propagation direction due to the quantum spin Hall effect of light. Introducing monochirality into the dielectric medium facilitates the differentiation of chiral enantiomers. Zhang et al. [47] found that chiral molecules are subject to chiral-selective lateral forces in opposite directions, which are not only used to identify chiral enantiomers but are also expected to achieve the separation of chiral enantiomers.

### 2.5. Comparison of the Four Methods

Benefiting from the LSPR effect of single nanoparticles and the mutual coupling between adjacent nanoparticles, chiral nanostructures based on nanoparticle self-assembly indicate a greater chiral optical response compared with chiral molecules. The preparation of chiral nanoparticles is mainly based on bottom-up chemical processes with broad application prospects in biochemical fields, i.e., chemical catalysis, disease diagnosis and treatment.

The nanophotonic platform based on the superchiral field utilizes the metal micro-nano structure to generate a near-field optical chirality that is stronger than the incident light. Interacting with the superchiral field, a stronger signal from the nearby chiral molecules is obtained. Although plasmonic nanostructures exhibit strong electric dipolarization and enhance near-field electric fields, their magnetic field enhancement is very limited. Therefore, it is crucial to design structures that enhance both electric and magnetic fields, especially magnetic components. On the other hand, as the nanostructures produce a strong CD response, it is not easy to effectively distinguish the contribution from the structure itself and the chiral analytes to the CD signal. At present, the literature [48] points out that this problem can be alleviated by using racemic nanostructures to detect chiral analytes.

Unlike superchiral structures directly generating CD signals, the PCCD induces the CD signals originating from Coulomb (dipole and multipole) interactions between achiral plasmonic nanostructures and chiral molecules. There is preliminary evidence that single-molecule PCCD is achievable, indicating that PCCD is promising for high-sensitivity measurements and even single-molecule detection [2,7].

All three of the above solutions involve the precise design and fabrication of nanostructures, which are avoided for platforms by using propagating surface plasmons. This scheme is compatible with existing surface plasmon resonance platforms based on the Kretschmann configuration and can identify chiral enantiomers with simple process and low cost. However, this solution is still in the theoretical exploration stage, and has no corresponding experimental results at present.

## 3. Applications

Benefiting from the nanoscale chirality in nanophotonic platforms, there has been a strong renaissance in the field of chirality. Enhanced chirality based on nanophotonics also has rich application scenarios, including chiral optical trapping [49,50], phase-change [51], catalysis [52,53], disease diagnosis [54] and treatment [55]. In this review, we mainly focus on the diagnosis and therapy of diseases.

### 3.1. Diagnosis of Disease

**Figure 8:**
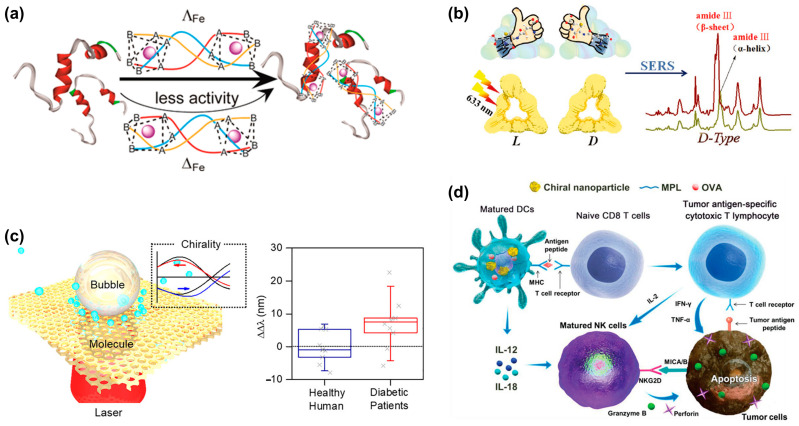
Applications of chiral nanostructures in clinical diagnosis and therapeutics. (**a**) Schematic representation of chiral metal supramolecular complexes that enantioselectively bind to amyloid-β (Aβ). Reprinted with permission from ref. [56]. Copyright 2014 American Chemical Society. (**b**) Synthesis of chiral triangular gold nanorings with SERS activity to detect Aβ42 proteins in AD patients. Reprinted with permission from ref. [54]. Copyright 2021 John Wiley and Sons. (**c**) Microbubble-induced accumulation and the detection of biomolecules on chiral plasmonic substrates. Reprinted with permission from ref. [58]. Copyright 2021 American Chemical Society. (**d**) Schematic diagram of the mechanism of chiral NPs in treating tumors. Reprinted with permission from ref. [59]. Copyright 2022 John Wiley and Sons.

As the most common form of dementia, Alzheimer’s disease (AD) affects more than 240,000 people worldwide. Studies have shown that the polymerization of amyloid-β (Aβ) peptides into amyloid fibrils is a key issue in the pathogenesis of AD. Therefore, the inhibition of Aβ aggregation is considered an attractive therapeutic and preventive strategy for AD treatment. In 2014, Li et al. [56] first reported their investigation on the enantioselective inhibition of Aβ aggregation. They designed two triple-helical binuclear metal supramolecular complexes as a new type of chiral amyloid-β inhibitor. As shown in Figure 8a, these metal complexes enantioselectively inhibit Aβ aggregation by targeting α/β-discordant extensions at early stages of aggregation. This study provides new insights into the chiral inhibition of Aβ aggregation and opens a new avenue for the design and screening of chiral drugs as Aβ inhibitors against AD. Subsequently, Tang’s research group [57] designed and prepared 3.3 nm L- and D-glutathione-stabilized gold nanoparticles (denoted as L3.3 and D3.3, respectively). Both chiral nanoparticles inhibit Aβ42 aggregation and pass the blood–brain barrier after intravenous administration without causing significant toxicity. Experimental results indicate that D3.3 has a greater binding affinity for Aβ42 and higher brain biodistribution compared with enantiomer L3.3, producing a stronger inhibitory effect on Aβ42 fibrillation in AD model mice. In addition to the method of directly using chiral signals for detection, Wang et al. [54] also synthesized L/D-Pt@Au triangular nanorings as a label-free surface-enhanced Raman spectroscopy platform to detect Aβ fibrils with ultra-high sensitivity, with the experimental results displayed in Figure 8b. This opens the means for the early diagnosis of diseases caused by protein misfolding using chiral plasmonic nanomaterials as ultrasensitive SERS substrates.

Diabetes is a common chronic disease, and its global incidence is increasing every year. Previous research has shown that elevated levels of many D-type metabolic molecules in urine are strongly related to diabetes [60,61]. Thus, monitoring abnormal chiral changes in urine metabolites may provide a promising approach for non-invasive diabetes diagnosis and specific clinical treatment. Chiral plasmonic nanostructures with superchiral fields are a promising method for the diagnosis of diabetes. Recently, Liu et al. designed a plasmonic moiré chiral metamaterial to generate photothermal microbubbles and superchiral fields simultaneously [58], as shown in Figure 8c. the Marangoni convection induced by microbubbles effectively drags biomolecules in the solution toward the laser spot to achieve enrichment. Finally, under the superchiral field, the chiral detection of glucose and lactate with concentrations as low as 100 pM is achieved. Meanwhile, a diagnostic accuracy of 84% was achieved on clinical urine samples from human patients. Therefore, this technology is expected to serve as an important means of first-line non-invasive screening and prognosis for prediabetes or diabetes and its complications.

Malignant tumors are a major disease that threaten human life and health. Although the treatment of tumors has made great progress through the efforts of scientists and clinicians, the high mortality rate has not yet been effectively controlled. The introduction of chiral nanomaterials is expected to provide a new means for the early diagnosis of cancer. Based on gold nanorod (NR) dimer assembly, Tang et al. [62] established a new biosensor for the ultrasensitive detection of prostate-specific antigen (PSA). The PSA aptamer (DNA1) and its complementary fragment (DNA2) were coupled to one side of the Au NRs, respectively. In the absence of PSA, Au NR-DNA1 hybridized with Au NR-DNA2, forming a dimer probe. With the addition of PSA, the aptamer tends to change its configuration to bind to PSA, which leads dimer dehybridization to form a single Au NR. The higher the PSA concentration, the weaker the CD intensity. The interaction between PSA and chiral sensors leads to changes in CD signal intensity, with potential applications in cancer diagnosis.

### 3.2. Treatment of Diseases

In addition to disease diagnosis, chiral nanostructures also has prospects for applications in cancer therapy. Figure 8d depicts a schematic diagram of the cancer immunotherapy mechanism using chiral NPs [59]. Chiral NPs stimulate the uptake of antigen by dendritic cells (DCs) and the crossover of the present antigenic peptide major histocompatibility complex class I (MHC I) to CD8 T cells. When CD8 T cells are activated, tumor necrosis factor-α (TNF-α) and interferon-γ (IFN-γ) are expressed, and antigenic peptides are specifically recognized through T cell receptors, which contribute to the elimination of tumor cells. At the same time, activated DCs and T cells express cytokines (IL-12, IL-18, and IL-2) to enhance the cytotoxic activity of natural killer (NK) cells.

Unlike the above-mentioned chiral nanoparticles, which directly stimulate dendritic cells, chiral plasmonic nanostructures are also used as an efficient chiral photosensitizer in photodynamic therapy (PDT) [63]. In 2017, Gao et al. [55] studied chiral nanostructures as label-free plasmonic-enhanced intracellular PDT agents for the ablation of tumor cells for the first time. Through DNA-based self-assembly process, they developed water-dispersible chiral PDT agents. Compared with traditional drugs, relatively low doses of chiral photosensitizers reach the intracellular reactive oxygen species threshold of PDT. Ultimately, tumors were completely ablated in mice treated with the developed chiral assemblies. Meanwhile, these nanostructures are non-toxic and have good biocompatibility, which is of great significance for future living biological applications.

Although the applications of nanophotonic chiral including detection, diagnosis, and treatment have been demonstrated, considering the excellent properties of chiral materials, their applications in the biomedical field are expected to be further developed.

## 4. Future Prospects and Challenges

This article reviews the chiral sensing mechanisms of various nanophotonic platforms and outlines the recent progress in four major methods in biosensing applications. We introduced four common chiral sensing platforms based on nanophotonics, including chiral nanostructure platforms, superchiral near-field sensing, plasmon-coupled circular dichroism, and surface plasmon resonance. We also elaborated on the applications of nano-optical chiral platforms in the detection and treatment of difficult diseases, such as Alzheimer’s disease, diabetes, and cancer.

Currently, chiral molecule identification and detection based on the nanophotonic platforms focus on distinguishing enantiomers and cannot evaluate the intrinsic CD response of the chiral analyte at present. However, with further theoretical analysis and experimental progress in nanophotonics, an analysis of the structure of the analyte itself is expected, which could enhance people’s understanding of the structure of biochemical molecules.

Although numerous chiral sensing methods have been achieved, creating a simpler method with a low cost is still an issue for large-scale applications in pharmaceutical industry. Although further in-depth research is needed, sensing platform based on surface plasmon resonance is a developing tendency for simple and low-cost chiral sensing. Also, in addition to the exploration of present applications in the diagnosis and therapy of diseases, more interactions between new biomarkers of diseases and chiral sensors need to be investigated in depth. We envision that the further development of chiral sensing technologies based on nanophotonics deepens the understanding of biomolecular structures and holds great promise in developing large-scale chiral-sensing platforms.

## Data Availability

Data sharing not applicable.
